# Twenty-Year Follow-Up of a Phase II Trial of Taxotere/Carboplatin/Herceptin in Patients With Metastatic HER2-Positive Breast Cancer

**DOI:** 10.1093/oncolo/oyad258

**Published:** 2023-09-19

**Authors:** Juan Luis Gomez Marti, Azadeh Nasrazadani, Ying Ding, Daniel Normolle, Adam M Brufsky

**Affiliations:** Department of Pathology, University of Pittsburgh School of Medicine, Pittsburgh, PA, USA; Department of Breast Medical Oncology, The University of Texas MD Anderson Cancer Center, Houston, TX, USA; Department of Biostatistics, University of Pittsburgh, Pittsburgh, PA, USA; Department of Biostatistics, University of Pittsburgh, Pittsburgh, PA, USA; UPMC Hillman Cancer Center, Department of Medicine, Division of Hematology-Oncology, Magee Women’s Hospital, Pittsburgh, PA, USA

**Keywords:** metastatic breast cancer, human epidermal growth factor receptor 2 (HER2), trastuzumab, chemotherapy, targeted therapy, survivorship

## Abstract

**Background:**

As the implementation of trastuzumab, several studies have investigated new agents and regimens to treat human epidermal growth factor 2-positive (HER2+) metastatic breast cancer (MBC). However, long-term survival analyses from HER2-targeted therapies are lacking. This is a 20-year follow-up study of a phase II trial that evaluated the activity and safety of docetaxel in combination with carboplatin and trastuzumab (TCH) in patients with HER2+ MBC.

**Materials and Methods:**

This is a follow-up of a phase II, single-arm, open-label study. The primary objective was the activity from the combination of TCH in terms of response rate (RR). Secondary objectives included the activity from TCH in terms of response duration (RD), time to progression (TTP), overall survival (OS), and percent one-year survival.

**Results:**

Between 11/1999 and 10/2002, 40 women were enrolled. Most patients (67.5%) had visceral metastasis on enrollment. After 2 decades and 3 months from first enrollment, there were 4 (10%) survivors (median follow-up of 3.2 years). The RR for complete response (CR) or partial response (PR) was 72.5%. The RR for CR was 42.5%. RD was 8 months. Median TTP was 10.8 months. Participants achieved a median OS of 39.8 months. Percent one-year survival was 92.5%.

**Conclusion:**

A subset of patients (10%) receiving trastuzumab in the metastatic setting have achieved long-term survival beyond 20 years.

## Teaser

Long-term survival analyses of HER2-targeted therapies are lacking. This is a 20-year follow-up study of a phase II trial that evaluated the activity and safety of docetaxel in combination with carboplatin and trastuzumab in patients with HER2+ metastatic breast cancer.

## Introduction

Human epidermal growth factor 2 (HER2) is overexpressed in approximately 15% of all breast cancer (BC) cases in the United States.^1^ Median overall survival (OS) of HER2-positive (HER2+) metastatic breast cancer (MBC) was less than 2 years prior to the advent of targeted therapies.^2^ These rapidly evolving treatments used alone or in combination, along with the discovery of novel biomarkers has translated into enormous progress with regard to outcomes,^3^ and description of long-term outcomes is feasible. In-depth analyses of cohorts who benefit from HER2-targeted therapies may aid in identification of clinicopathologic and molecular features with therapeutic-rognostic significance. In the present study, we report the long-term results of a phase II, single-arm, open-label clinical trial of carboplatin and trastuzumab (TCH) in women with advanced HER2 + MBC after 20 years of follow-up. A small cohort of long-term survivors has been identified and characterized.

## Materials and Methods

UPCI 99-058 was a phase II single-arm, open-label clinical trial of Taxotere (docetaxel) in combination to carboplatin and Herceptin (trastuzumab) in patients with HER2+ MBC. Forty patients were enrolled at the University of Pittsburgh Cancer Institute (UPCI). The study was conducted in accordance with the Declaration of Helsinki. Patients were provided with written informed consent prior to registering in the study.

The primary objective was RR. Secondary objectives were response duration (RD), time to progression (TTP), OS, percent one-year survival, and safety. Definition of response objectives and inclusion/exclusion criteria can be found in the study protocol. The present study was initiated in 1999, and therefore, measurement of lesions following RECIST was not performed.

Patient accrual followed a Simon 2-step study design. Kaplan-Meier methods were used to assess TTP and OS. Data cutoff for TTP and OS was February 18, 2020. Chi-squared tests with Yates’ correction were used to compare the distribution of clinically meaningful categorical variables. Statistical tests were performed 2-sided with an α of <0.05 to be considered statistically significant.

The study protocol (IRB#0411034) was approved by the institutional review board (IRB) and conducted in accordance with the Declaration of Helsinki. Patients were provided with written informed consent prior to registering in the study.

## Results

### Outcomes

As of August 2019, a total of 4 (10%) patients were alive and on follow-up. The median follow-up was 3.2 years. The cause of death was attributed to malignant disease in 18 participants (50%), to other causes in 3 patients (8.3%), and unknown in 15 (42%) patients.

The response rate (RR) for complete response (CR) or partial response (PR) (CR + PR) was 72.5% (*n* = 29). The RR for CR was 42.5% (*n* = 17). The median RD for CR + PR was 8. 05 months (95% CI, 116-1741), and the median duration for CR was 9.34 months (95% CI, 117-1741; Table 1).

**Table 1.** Primary objectives.

	*N*	Mean	Median	Range
Complete or partial response	29	404.3	245	116.0, 1741.0
Complete response	17	467.8	284	117.0 1741.0

Median TTP was 10.78 months (95% CI, 263-525) (Supplementary Table S1). Participants achieved a median OS of 39.78 months (95% CI, 791-2415; Supplementary Table S1). Percent one-year survival was 92.5% (37 out of 40 participants survived for at least a year).

### Patients

Enrollment occurred between 11/1999 and 10/2002. We characterized the subset of survivors as a separate group for descriptive analysis. One patient survived for a period of 16.97 years after enrollment and died a year before the 20-year follow-up; the participant was included in the group of survivors for the descriptive analysis. Age, ECOG status, and estrogen/progesterone receptor (ER/PR) status were similarly balanced between groups. Patient characteristics are summarized in Table 2.

**Table 2.** Patient characteristics at baseline.

	Total	Non-survivors (35)	Survivors (5)
Age, median years (range)	56.5 [33-79]	57.6	57.1
Age category			
<50		10 (29%)	
>50		25 (71%)	5 (100%)
Follow-up duration (years)	2.77 [0.76,17.03]		
Ethnicity			
White	38 (95%)	33 (94%)	5 (100%)
African American	1 (2.5%)	1 (2.9%)	
Asian Indian/Pakistani	1 (2.5%)	1 (2.9%)	
ECOG performance status			
0	38 (95%)	33 (94%)	5 (100%)
1	2 (5%)	2 (6%)	
Tumor location			
UOQ	23 (57.5%)	22 (63%)	1 (20%)
NOS	7 (1.75%)	6 (17%)	1 (20%)
Central	4 (1%)	4 (11%)	
UIQ	3 (0.75%)	1 (2.9%)	2 (40%)
LOQ	2 (0.5%)	1 (2.9%)	1 (20%)
Axillary tail	1 (0.25%)	1 (2.9%)	
Histology type			
Adenocarcinoma, MET, NOS	17	15 (43%)	2 (40%)
Infiltrating ductal carcinoma, MET	9	8 (23%)	
Adenocarcinoma, NOS	6	4 (11%)	2 (40%)
Infiltrating ductal and lobular carcinoma	4	4 (11%)	
Adenoma, NOS	1	1 (2.9%)	
Infiltrating ductal carcinoma	1	1 (2.9%)	1 (20%)
Intraductal carcinoma noninfiltrating, NOS	1	1 (2.9%)	
Paget’s disease and infiltrating ductal carcinoma	1	1 (2.9%)	
ER status			
Positive	9	8 (23%)	1 (20%)
Negative	31	27 (77%)	4 (80%)
Metastases at time of diagnosis???			
Visceral	29	26 (74%)	3 (60%)
Liver	19	18 (51%)	1 (20%)
Lung	16	13 (37%)	3 (60%)
Bone	5	5 (14%)	
Lymph nodes	14	13 (37%)	1 (20%)
Chest wall	4	2 (5.7%)	2 (40%)
New brain (upon initiation of study) this might need to be a separate category of table	7	6 (17%)	1 (20%)
Metastatic sites			
≤2	34	29 (83%)	5 (100%)
>2	5	5 (14%)	
Previous lines of chemotherapy	16 (46%)	2 (40%)	
0	27 (67.5%)	24 (69%)	3 (60%)
1	9 (22.5%)	7 (2%)	2 (40%)
2	4 (10%)	4 (11%)	
Type previous chemotherapy (if any)			
CMF	2 (5%)	2 (6%)	
Taxol	5 (12.5%)	5 (14%)	
AC	11 (27.5%)	9 (26%)	2 (40%)
SERM	11 (27.5%)	10 (29%)	1 (20%)
Prior radiation			
Locoregional	11 (27.5%)	8 (22.8%)	3 (60%)
Metastatic	4 (10%)	3 (8.6%)	1 (20%)
Brain		1 (2.9%)	1 (20%)
Lumbar		1 (2.9%)	
Iliac		1 (2.9%)	
Unknown	1 (2.5%)	1 (2.9%)	

Abbreviations: ECOG: Eastern Cooperative Oncology Group; UOQ: upper outer quadrant; NOS: not otherwise specified; UIQ: upper inner quadrant; LOQ: lower outer quadrant; CMF: cytoxan + methotrexate + 5-fluoracil; AC: adriamycin + cytoxan; SERM: selective estrogen receptor modulator.

Most participants had visceral metastasis on enrollment (*n* = 27; 67.5%); the most frequent site being the liver (*n* = 19; 47.5%). The frequency of liver involvement in non-survivors and survivors was 51% (*n* = 18) and 20% (*n* = 1), respectively (*P* = .4). On the other hand, degree of lung involvement between nonsurvivors and survivors was 37% (*n* = 13) and 60% (*n* = 3), respectively (*P* = .6). All survivors (100%) had ≤2 metastatic sites involved, whereas this only accounted for 83% (*n* = 29) of nonsurvivors. Before enrolling, 9 (22.5%) and 4 (10%) patients had undergone 1 and 2 lines of chemotherapy, respectively. Among the group of nonsurvivors, 2 (6%) patients had received cytoxan + methotrexate + 5-fluoracil, 5 (14%) patients had received taxol, and 9 (26%) had been exposed to adriamycin + cytoxan (AC). On the other hand, survivors only were exposed to AC prior to enrollment (*n* = 2, 40%). A total of 14 patients (35%) had previously received radiotherapy (RT) prior to entering the study, with 11 patients (27.5%) receiving locoregional contrast tomagraphy (CT scan) and 4 patients (10%) receiving RT to distant sites (Table 2). Among the group of survivors, 3 (60%) had received locoregional, and 1 (20%) patient had received metastatic brain radiation. In the group of nonsurvivors, 8 (22.8%) received locoregional, and 3 (8.6%) received metastatic radiation (Table 2). To our knowledge, survivors did not receive any additional HER2-targeted therapy.

### Adverse Events and Laboratory Assessments

Treatment-related AE are shown in Supplementary Tables S2-S3. On long-term follow-up (>10 years), we only found trastuzumab-related AEs in one patient (20% of survivors).

## Discussion

Trastuzumab continues to be the backbone of therapy in the first-line setting for management of HER2+ MBC. When given in combination with a second anti-HER2-directed agent, pertuzumab, a considerable 18.5-month progression-free survival (PFS) is achieved, as observed in CLEOPATRA.^4^ In BCIRG 007, single use of trastuzumab in combination with docetaxel demonstrated median TTP of 11 months, CR rate of 18%, and median OS of 37 months.^5^ In terms of TTP and OS, these results were similar to the findings reported herein. While significant benefit has consistently been observed with Herceptin, long-term studies reporting activity and safety were needed.

To our knowledge, this study provides the longest follow-up on trastuzumab when utilized in the metastatic setting and is the first to identify and clinically characterize a cohort of survivors after 2 decades. Use of TCH for women with HER2+ MBC resulted in a 10% OS with median OS of 39.78 months, median TTP of 10.78 months, and a PR and CRR of 72.5% and 42.5%, respectively. In terms of response follow-up to dual HER2-blockade, the end-of-study results from CLEOPATRA reported a median OS of 57.1 months in contrast to the 40.8 months of median OS in the placebo arm (trastuzumab/docetaxel alone) (HR 0.69; 95% CI, 0.58-0.82). The updated 8-year follow-up results showed a 37% OS in contrast to a 23% OS achieved in the placebo arm.^4^ Here, the 8-year OS follow-up cohort would be of 22.5% and 10-year OS of 12.5%.

In a 5-year follow-up study of trastuzumab in combination with pertuzumab and paclitaxel, 86% of participants achieved a 6-month PFS, with a median of 24.2 months.^6^ The median OS for patients with no prior metastatic chemotherapy was 39.7 months. Our results indicate a similar OS despite the use of a single HER2 inhibitor. The results from the present study are also inline with a previous trial that evaluated the use of weekly paclitaxel/carboplatin with or without trastuzumab after trastuzumab use. The study reported a CRR of 25%, time to progression of 14.2 months, and a median OS of 32.2 months, among those patients who were able to receive triplet therapy after trastuzumab use.^7^

AE at long-term follow-up were similar to those previously reported and are, therefore, not discussed in detail.^5,8^

Our study contains limitations that are acknowledged. Cause of death was not attributed to a specific cause in 42% of participants, as this data were not available. As a result, conclusions regarding cancer-specific mortality could not be drawn. To our knowledge, patients did not receive any additional targeted therapies other than Herceptin.

Within our cohorts, all patients had an aggressive disease course marked by the presence of stage IV disease on enrollment. Remarkably, the presence of visceral and even brain metastases did not lead to a poorer outcome in some patients, pointing out the need for enhanced characterization of tumors that might be highly sensitive to HER2 inhibition. Identification of additional genomic biomarkers associated with long-term survival may guide therapy individualization to allow more aggressive management of patients anticipated to respond poorly to standard of care therapies. Conversely, enhanced understanding of patients achieving durable responses to therapy may aid in de-escalation approaches. Further follow-up from recent phase II and phase III clinical trials are awaited.

## Supplementary Material

oyad258_suppl_Supplementary_Tables

## Data Availability

The data underlying this article will be shared on reasonable request to the corresponding author.
