# Supplementary material for: Twenty-Year Follow-Up of a Phase II Trial of Taxotere/Carboplatin/Herceptin in Patients With Metastatic HER2-Positive Breast Cancer
Source: Oncologist. 2023 Sep 19;28(11):e1123–6. doi: 10.1093/oncolo/oyad258 (PMC10628556; doi:10.1093/oncolo/oyad258)
Supplement: oyad258_suppl_Supplementary_Tables [file oyad258_suppl_supplementary_tables.docx]

**Supplementary Table 1.** Secondary objectives.

|  | **N** | **N events** | **median** | **95% CI for median** |
| --- | --- | --- | --- | --- |
| Time-to-progression (day) | 40 | 29 | 328 | [263, 525] |
| Overall survival (day) | 40 | 36 | 1210 | [791, 2415] |

**Supplementary Table 2.** Adverse events.

| **Toxicity** | **Category** | **Non-Survivors** | **Survivors** | **Total** |
| --- | --- | --- | --- | --- |
| Neutropenia | Hematologic | 34 (97.1%) | 5 (100%) | 39 (97.5%) |
| Anemia | Hematologic | 30 (85.8%) | 5 (100%) | 35 (87.5%) |
| Fatigue, agitation, confusion, disorientation, hallucinations, coma, seizures | Neurologic | 27 (77.1%) | 5 (100%) | 32 (80%) |
| Thrombocytopenia | Hematologic | 26 (74.3%) | 4 (80%) | 30 (75%) |
| Leukopenia | Hematologic | 27 (77.1%) | 2 (40%) | 29 (72.5%) |
| Nausea, upset stomach, dry heaves | Gastrointestinal | 22 (62.9%) | 5 (100%) | 27 (67.5%) |
| Alopecia/Hair Loss | Other | 21 (60%) | 4 (80%) | 25 (62.5%) |
| Diarrhea | Gastrointestinal | 19 (54.3) | 1 (20%) | 20 (50%) |
| Other miscellaneous condition | Other﻿ Other | 16 (45.7%) | 2 (40%) | 18 (45.5%) |
| Peripheral Edema | Cardiovascular | 12 (34.3%) | 4 (80%) | 16 (40%) |
| Fever | Flu-like Symptoms | 14 (40%) |  | 14 (35%) |
| Anorexia | Gastrointestinal | 13 (37.1%) | 1 (20%) | 14 (35%) |
| Skin rash, dry skin | Other | 12 (34.3%) | 1 (20%) | 13 (32.5%) |
| Altered Taste, metallic taste | Gastrointestinal | 11 (31.4%) | 1 (20%) | 12 (30%) |
| Myalgias/Arthralgias | Flu-like Symptoms | 10 (28.6%) | 2 (40%) | 12 (30%) |
| Vomiting | Gastrointestinal | 10 (28.6%) | 2 (40%) | 12 (30%) |
| Paresthesia | Neurologic | 7 (20%) | 3 (60%) | 10 (25%) |
| Other neurologic condition | Neurologic | 7 (20%) | 1 (20%) | 8 (20%) |
| Infection | Other | 6 (17.1%) | 1 (20%) | 7 (17.5%) |
| Stomatitis ulcers | Gastrointestinal | 5 (14.2%) | 2 (40%) | 7 (17.5%) |
| AP Increase | Liver Function Test | 6 (17.1%) | 1 (20%) | 7 (17.5%) |
| AST Increase | Liver Function T | 6 (17.1%) |  | 6 (15%) |
| Other EENT Condition | Eye, Ear, Nose, Throat | 6 (17.1%) |  | 6 (15%) |
| Vertigo Dizziness | Neurologic | 6 (17.1%) |  | 6 (15%) |
| LDH Increase | Liver Function Test | 6 (17.1%) |  | 6 (15%) |
| Hyperglycemia | Metabolic | 5 (14.3%) | 1 (20%) | 6 (15%) |
| Increased BUN | Genitourinary | 5 (14.3%) |  | 5 (12.5%) |
| Hypochloremia | Electrolyte | 5 (14.3%) |  | 5 (12.5%) |
| Indigestion/Heartburn | Gastrointestinal | 5 (14.3%) |  | 5 (12.5%) |
| Miscellaneous throat conditions | Eye, Ear, Nose, Throat | 4 (11.4%) | 1 (20%) | 5 (12.5%) |
| Other flu-like condition | Flu-like Symptoms | 4 (11.4%) | 1 (20%) | 5 (12.5%) |
| Pain | Other | 4 (11.4%) | 1 (20%) | 5 (12.5%) |
| Shortness of breath | Pulmonary | 2 (5.7%) | 3 (60%) | 5 (12.5%) |
| Other GI Condition | Gastrointestinal | 4 (11.4%) |  | 4 (10%) |
| Chills/Rigor | Flu-like Symptoms | 3 (8.6%) | 1 (20%) | 4 (10%) |
| Neuro-motor weakness | Neurologic | 2 (5.7%) | 2 (40%) | 4 (10%) |
| Pain/Discomfort | Musculoskeletal | 3 (8.6%) |  | 3 (7.5%) |
| Constipation | Gastrointestinal | 3 (8.6%) |  | 3 (7.5%) |
| Cough | Pulmonary | 3 (8.6%) |  | 3 (7.5%) |
| Mood: Depression, anxiety, suicidal | Neurologic | 3 (8.6%) |  | 3 (7.5%) |
| Other cardiac condition | Cardiovascular | 3 (8.6%) |  | 3 (7.5%) |
| Hypocalcemia | Metabolic | 2 (5.7%) | 1 (20%) | 3 (7.5%) |
| Weight Loss | Weight | 2 (5.7%) | 1 (20%) | 3 (7.5%) |
| Miscellaneous nose conditions | Eye, Ear, Nose, Throat | 2 (5.7%) | 1 (20%) | 3 (7.5%) |
| Facial flushing | Flu-like Symptoms | 2 (5.7%) | 1 (20%) | 3 (7.5%) |
| Headache | Neurologic | 2 (5.7%) | 1 (20%) | 3 (7.5%) |
| Miscellaneous eye conditions | Eye, Ear, Nose, Throat | 2 (5.7%) |  | 2 (5%) |
| Hyperkalemia | Electrolyte | 2 (5.7%) |  | 2 (5%) |
| Hypercarbia | Electrolyte | 2 (5.7%) |  | 2 (5%) |
| ALT Increase | Liver Function Test | 2 (5.7%) |  | 2 (5%) |
| Hypoproteinemia | Metabolic | 2 (5.7%) |  | 2 (5%) |
| Hypoalbuminemia | Metabolic | 2 (5.7%) |  | 2 (5%) |
| Thrombocytosis | Hematologic | 1 (2.9%) | 1 (20%) | 2 (5%) |
| Other GU condition | Genitourinary | 1 (2.9%) | 1 (20%) | 2 (5%) |
| Angina | Cardiovascular | 1 (2.9%) |  | 1 (2.5%) |
| Miscellaneous ear conditions | Eye, Ear, Nose, Throat | 1 (2.9%) |  | 1 (2.5%) |
| Hypercalcemia | Metabolic | 1 (2.9%) |  | 1 (2.5%) |
| Increased creatinine | Genitourinary | 1 (2.9%) |  | 1 (2.5%) |
| Weight gain | Weight | 1 (2.9%) |  | 1 (2.5%) |
| Dysrhythmias | Cardiovascular | 1 (2.9%) |  | 1 (2.5%) |
| Hyponatremia | Electrolyte | 1 (2.9%) |  | 1 (2.5%) |
| Hypokalemia | Electrolyte | 1 (2.9%) |  | 1 (2.5%) |
| Hypomagnesemia | Metabolic | 1 (2.9%) |  | 1 (2.5%) |
| Other Musculo-skeletal condition | Musculoskeletal | 1 (2.9%) |  | 1 (2.5%) |
| Thrombosis | Circulatory | 1 (2.9%) |  | 1 (2.5%) |
| AllergyAnaphylaxis | Other | 1 (2.9%) |  | 1 (2.5%) |
| Hypocarbia | Electrolyte | 1 (2.9%) |  | 1 (2.5%) |
| Rectal bleeding | Gastrointestinal | 1 (2.9%) |  | 1 (2.5%) |
| Cramping | Gastrointestinal | 1 (2.9%) |  | 1 (2.5%) |
| Dehydration | Other | 1 (2.9%) |  | 1 (2.5%) |
| Other hematologic Condition | Hematologic | 1 (2.9%) |  | 1 (2.5%) |
| Cerebellar: Incoordination, intention tremor, ataxia, nystagmus | Neurologic | 1 (2.9%) |  | 1 (2.5%) |

WBC= White blood cell; EENT= Eye, Ear, Nose, Throat; LDH= Lactate dehydrogenase; BUN= Blood urea nitrogen; GI= Gastrointestinal; ALT= Alanine aminotransferase; GU= Genitourinary; AP= Alkaline Phosphatase; AST= Aspartate aminotransferase

**Supplementary Table 3.** Grades 3 and 4 adverse events.

| **Toxicity** | **Category** | **Non-Survivors** | **Survivors** | **Total** |
| --- | --- | --- | --- | --- |
| **Grade 3** |  |  |  |  |
| Leukopenia | Hematologic | 21 (60%) | 2 (40%) | 23 (57.5%) |
| Neutropenia | Hematologic | 18 (51.4%) | 3 (60%) | 21 (52.5%) |
| Thrombocytopenia | Hematologic | 9 (25.7%) | 1 (20%) | 10 (25%) |
| Anemia | Hematologic | 4 (11.4%) |  | 4 (10%) |
| Fatigue, agitation, confusion, disorientation, hallucinations, coma, seizures | Neurologic | 3 (8.5%) | 1 (20%) | 4 (10%) |
| Infection | Other | 3 (8.5%) |  | 3 (7.5%) |
| Other miscellaneous condition | Other | 2 (5.7%) |  | 2 (5%) |
| Fever | Flu-like Symptoms | 2 (5.7%) |  | 2 (5%) |
| Other Flu-like condition | Flu-like Symptoms | 1 (2.9%) |  | 1 (2.5%) |
| Other hematologic condition | Hematologic | 1 (2.9%) |  | 1 (2.5%) |
| Other cardiac condition | Cardiovascular | 1 (2.9%) |  | 1 (2.5%) |
| Nausea, upset stomach, dry heaves | Gastrointestinal |  | 1 (20%) | 1 (2.5%) |
| Vomiting | Gastrointestinal |  | 1 (20%) | 1 (2.5%) |
| Diarrhea | Gastrointestinal |  | 1 (20%) | 1 (2.5%) |
| Skin rash, dry skin | Other |  | 1 (20%) | 1 (2.5%) |
| **Grade 4** |  |  |  |  |
| Diarrhea | Gastrointestinal | 0 | 1 (20%) | 1 |
| Leukopenia | Hematologic | 14 (40%) | 2 (40%) | 16 (40%) |
| Thrombocytopenia | Hematologic | 5 (14.3%) | 0 | 5 (12.5%) |
| Fever | Flu-like Symptoms | 1 (2.9%) | 0 | 1 (2.5%) |
| Infection | Other | 1 (2.9%) | 0 | 1 (2.5%) |
| Neutropenia | Hematologic | 28 (80%) | 5 (100%) | 33 (82.5%) |
| Other cardiac condition | Cardiovascular | 1 (2.9%) | 0 | 1 (2.5%) |
